# Investigating the Intersection of Policing and Public Health

**DOI:** 10.1371/journal.pmed.1001571

**Published:** 2013-12-10

**Authors:** Scott Burris, Stephen Koester

**Affiliations:** 1 Beasley School of Law and Department of Public Health, Temple University, Philadelphia, Pennsylvania, United States of America; 2 Department of Anthropology, and Department of Health and Behavioral Sciences, University of Colorado Denver, Denver, Colorado, United States of America

## Abstract

In an accompanying Perspective, Scott Burris and Stephen Koester discuss the association between punitive policies and health inequalities and argue for more research focusing on policing and health.

*Please see later in the article for the Editors' Summary*

**Linked Research Article**

This Perspective discusses the following new study published in *PLOS Medicine*:

Hayashi K, Small W, Csete J, Hattirat S, Kerr T (2013) Experiences with policing among people who inject drugs in Bangkok, Thailand: a qualitative study. PLoS Med 10(12): e1001570. doi:10.1371/journal.pmed.1001570

Using thematic analysis, Kerr and colleagues document the experiences of policing among people who inject drugs in Bangkok and examine how interactions with police can affect drug-using behaviors and health care access.

The study by Kerr and colleagues [1] in this week's *PLOS Medicine* adds to the evidence from many single-site qualitative and mixed-methods studies, from countries rich and poor, that police misconduct, ranging from interfering with harm reduction programs to extortion, beatings, and rape, exacerbates the risk of HIV and other diseases spread through injection drug use [2],[3]. Given Thailand's much criticized record of compulsory drug treatment of dubious therapeutic validity [4], the study's description of a rise in ethically and legally unconstrained urine testing is particularly important: it takes more than legislation characterizing drug users as “patients” to instill a therapeutic mentality in law enforcement agencies historically trained and incentivized to get tough on drugs.

Many police officers and agencies are dedicated to promoting public welfare and safety through appropriate legal means, but there is no mystery as to why and how abuse happens even in professionalized and well-run organizations. For reasons good and bad, police managers set arrest quotas or otherwise demand or incentivize arrests or mandatory treatment [5],[6]. More fundamentally, the punitive laws and the social attitudes and power relations that they reflect give police permission to use coercive means to manage—and too often exploit—people who live at the intersection of legal and social marginalization [7].

It is fair to say that researchers cannot define the rate of this sort of misconduct, but also that researchers and human rights advocates seem to be able to uncover abuse just about everywhere they look. Stratospheric incarceration rates, disparately high rates of HIV among the poor and policed, and epidemics that cannot be prevented for lack of proven interventions, such as syringe exchange and opioid replacement therapy, stand as ample population-level evidence of the harms of punitive laws and policing practices. Twenty years ago, the connection between policing and HIV was invisible. Research has exposed it and put it on the policy agenda [8]—but there is more to do to bring policing and public health into effective partnership.

## Policing and Health Inequities

Studies such as Kerr and colleagues' document how laws and legal practices both reinforce social inequalities and act as a mechanism for transforming social status into unequal and inequitable distributions of health [9]. In a time of rising and spreading inequality, it is not enough for research to document inequality's many pathological effects at the distal end of the causal chain [10]. The challenge is rather to lay bare the powerful relationship between punitive laws and legal practices and the deeper determinants of health. Health research can and should engage the relationship between punitive social control practices and global economic policies including trade liberalization and reductions in state-supported public services that have marginalized increasing numbers of the world's population while enabling a small fraction to become incomprehensibly rich [11]. Ethnographic and mixed methods research incorporating this perspective can address the health processes and consequences associated with the imperative in inequitable societies to regulate and manage the poor [12]. Nor are researchers limited to analyses of structural inequality and qualitative studies of their expression in human misery. Quasi-experimental methods using large datasets of laws, mediators, and outcomes varying over space and time can credibly demonstrate the causal relationship between punitive policies and poorer levels and distributions of health, and delineate more clearly the pathways along which they operate [13],[14]. In the spirit of Geoffrey Rose, research can uncover the causes of the differing incidence of health and social problems in populations exposed to high inequality and oppression compared to those that are not [15].

## A Research Agenda for Healthy Policing

Research focusing on policing and health has supported practical efforts to deal with common drivers of unhealthy police conduct at a time when the role of police as agents of health has grown both in size and recognition [16],[17]. The War on Drugs, and social disinvestment in mental health, education, and other social services, have brought unwell people in huge numbers into courts, jails, and prisons. More or less genuine and effective therapeutic responses have followed. Police are triaging psychosis on a daily basis, providing outreach to the homeless, and making decisions about whether to manage drug abuse as a crime, an illness, or a pastime. In programs like Seattle's Law Enforcement Assisted Diversion (LEAD), officers can divert drug users to treatment at the time of arrest. Courts are now providers or managers of treatment and social services for drug users, veterans, fractured families, wounded children, and the mentally ill. The true therapeutic value of these programs, the actual willingness and capacity of law enforcement agents to deliver them properly, and their health outcomes are all questions that should be high on the research agenda. Research like this will support police, who often receive little or no additional funding, training, or other professional support to do the hard new jobs assigned them. It is equally important to study how well the targets of punitive policing are mobilizing to resist and reform laws and police behavior. Drug user unions, sex-worker collectives, and legal services can all provide means for those targeted by police misconduct to resist mistreatment and reshape risk environments [18],[19].

Finally, nascent efforts to bridge the institutional and cultural gaps between law enforcement, public health, harm reduction, and drug users deserve greater attention and support. It is evident from experiences with marijuana in the US, drug depenalization in Portugal, and the adoption of “four pillars” approaches in places like Switzerland and Vancouver that local and national drug policy and practice can change. Serious proposals for global reform are on the table [20]. It is also obvious, however, that making drugs legal does not make them safe for all users in all circumstances. As the experience with alcohol has taught, law enforcement has a role in limiting the harms of legal as well as illegal drugs [21]. Moving to a “public health approach” will not eliminate the need for better integration of policing and public health, nor for research that can inform it.
